# Association between Inflammatory Infiltrates and Isolated Monosomy 22/del(22q) in Meningiomas

**DOI:** 10.1371/journal.pone.0074798

**Published:** 2013-10-01

**Authors:** Patrícia Henriques Domingues, Cristina Teodósio, Álvaro Otero, Pablo Sousa, Javier Ortiz, María del Carmen García Macias, Jesús María Gonçalves, Ana Belén Nieto, María Celeste Lopes, Catarina de Oliveira, Alberto Orfao, Maria Dolores Tabernero

**Affiliations:** 1 Centre for Neurosciences and Cell Biology and Faculty of Pharmacy, University of Coimbra, Coimbra, Portugal; 2 Centre for Cancer Research (CIC-IBMCC; CSIC/USAL; IBSAL) and Department of Medicine, University of Salamanca, Salamanca, Spain; 3 Neurosurgery Service, University Hospital of Salamanca, Salamanca, Spain; 4 Pathology Service, University Hospital of Salamanca, Salamanca, Spain; 5 Molecular Pathology Service, Centre for Cancer Research (CIC-IBMCC; CSIC/USAL), Salamanca, Spain; 6 Instituto de Estudios de Ciencias de la salud de Castilla y León (IECSCYL-IBSAL) and Research Unit of the University Hospital of Salamanca, Salamanca, Spain; UCLA, United States of America

## Abstract

Meningiomas contain highly variable levels of infiltrating tissue macrophages (TiMa) and other immune cells. In this study we investigated the potential association between the number and immunophenotype of inflammatory and other immune cells infiltrating the tumor as evaluated by multiparameter flow cytometry, and the clinico-biological, cytogenetic and gene expression profile (GEP) of 75 meningioma patients. Overall, our results showed a close association between the amount and cellular composition of the inflammatory and other immune cell infiltrates and the cytogenetic profile of the tumors. Notably, tumors with isolated monosomy 22/del(22q) showed greater numbers of TiMa, NK cells and (recently)-activated CD69^+^ lymphocytes versus meningiomas with diploid and complex karyotypes. In addition, in the former cytogenetic subgroup of meningiomas, tumor-infiltrating TiMa also showed a more activated and functionally mature phenotype, as reflected by a greater fraction of CD69^+^, CD63^+^, CD16^+^ and CD33^+^ cells. GEP at the mRNA level showed a unique GEP among meningiomas with an isolated monosomy 22/del(22q) versus all other cases, which consisted of increased expression of genes involved in inflammatory/immune response, associated with an M1 TiMa phenotype. Altogether, these results suggest that loss of expression of specific genes coded in chromosome 22 (e.g. *MIF*) is closely associated with an increased homing and potentially also anti-tumoral effect of TiMa, which could contribute to explain the better outcome of this specific good-prognosis cytogenetic subgroup of meningiomas.

## Introduction

Meningiomas are usually considered to be benign central nervous system tumors, on both histopathological [1] and clinical [2] grounds. Despite this, a significant fraction of all meningiomas will eventually relapse with a negative impact in patient outcome [2]. In recent years, tumor cytogenetics has emerged as the most relevant prognostic factor, together with tumor histopathology/grade and patient age and sex [3], [4]. Whereas cases displaying complex karyotypes, particularly monosomy 14 in association with del(1p), display a dismal outcome [5], tumors with isolated monosomy 22/del(22q) show a particularly good prognosis, the molecular basis of such clinical behavior remaining to be fully understood [3], [5], [6].

Although tumor histopathology and tumor behavior are, at least in part, related to tumor cytogenetics [2], [7], they might also be influenced by specific changes in the tumor microenvironment [8], [9]. In this regard, we have recently reported the existence of variable levels of infiltration of meningiomas by inflammatory and other immune cells [10]. CD45^+^ inflammatory cells that infiltrated meningiomas mainly included tissue macrophages (TiMa) with an HLA-DR^+^CD14^+^CD45^+^CD68^+^CD16^−/+^CD33^−/+^ phenotype and a high phagocytic/endocytic activity, together with a smaller population of cytotoxic lymphocytes, mostly CD8^+^ T cells and NK-cells [10]. Previous studies in other tumor types such as melanoma and colorectal cancer [11], [12], have shown that infiltration by immune cells is associated with specific features of the disease, including a better outcome. In meningiomas, previous reports indicate that the type and level of infiltrating inflammatory cells are both associated with the histopathological features of the tumor [9], [10], [13]. However, so far no study has investigated the potential association between tumor infiltrates of inflammatory and other immune cells, and other features of the disease including cytogenetics.

In this study we investigated the association between the cellular composition and protein expression profiles of meningiomas as analyzed by multiparameter flow cytometry, and the clinico-biological, genetic and mRNA gene expression profiling features of the disease. Our results indicate that the presence of inflammatory infiltrates of antigen presenting cells (TiMa) and lymphocytes is clearly associated with tumors displaying isolated monosomy 22/del(22q), which could contribute to explain the better outcome of this specific cytogenetic subgroup of meningiomas.

## Materials and Methods

### Patients and samples

Overall, 78 tumor samples from 75 patients (20 males and 55 females; mean age of 60±14 years; range: 23 to 84 years) diagnosed with meningioma at the Neurosurgery Service of the University Hospital of Salamanca (Salamanca, Spain), were analyzed in this study. According to the WHO criteria [2] 64 (82%) tumors were benign/grade I meningiomas, 11 (14%) were atypical/grade II tumors and 3 (4%) were grade III meningiomas. Fresh tumor tissue samples and ethylenediamine tetraacetic acid (EDTA)-anticoagulated peripheral blood (PB) samples were obtained in parallel from each patient, after written informed consent was given according to the guidelines of the local Ethics Committee. Tumor tissue samples were frozen in liquid nitrogen immediately after surgical removal, whereas PB samples were immediately processed as described below.

Brain edema was found in 37/75 (49%) patients. According to its extension, it was evaluated as light (smaller or equal to the volume of the tumor) in 11 cases (15%), moderate (doubling the volume of the tumor) in 16 cases (21%) and severe (more than twice the volume of the tumor) in 10 cases (13%). The most relevant clinical, histopathological and cytogenetic features of the tumors analyzed by flow cytometry and gene expression profiling are summarized in Table S1. The study was approved by the local Ethics Committee and Institutional Review Board of the University Hospital of Salamanca. At the moment of closing this study 6/75 patients had relapsed (8%) after a median follow-up of 48 months (range: 1 to 238 months). From these relapsed cases, all except one underwent complete tumor resection and three were submitted to radiotherapy; 4 were benign/grade I meningiomas, 1 a grade II tumor and the other case was a grade III meningioma.

### Multiparameter flow cytometry (MFC) immunophenotyping

For the immunophenotypic flow cytometry analyses, single tumor cell suspensions were obtained from 51 freshly-frozen meningioma samples (46 WHO grade I, 3 WHO grade II and 2 WHO grade III tumors), through conventional mechanical disaggregation procedures [14], [15] in phosphate buffered saline (PBS) containing 10% fetal bovine serum (FBS; Invitrogen, Carlsbad, CA, USA), 1% bovine serum albumin (BSA) (Sigma, St. Louis, MO, USA) and 2 mM EDTA (Merck, Darmstadt, Germany). Thawed meningioma cells were stained for 30 min at 4°C in the darkness with the following monoclonal antibodies (mAb): CD2- fluorescein isothiocyanate (FITC), CD13- phycoerythrin (PE), CD14-PE, CD33-PE, CD58-PE, CD69-PE, HER2/neu-PE and HLA-DR-FITC, purchased from Becton/Dickinson (BD) Biosciences (BDB, San Jose, CA, USA); CD22-FITC, CD37-FITC, CD53-PE, CD55-FITC, CD81-PE and CD99-PE from BD Pharmigen (San Diego, CA, USA); CD9-FITC, CD16-FITC, CD63-FITC and HLA-I-FITC purchased from Beckman/Coulter (Fullerton, CA, USA); CD44-PE and CD59-FITC obtained from Immunostep SL (Salamanca, Spain); Bcl2-FITC and CD45-Pacific Blue (PacB) purchased from DAKO (Glostrup, Denmark); CD38-FITC from Cytognos SL (Salamanca, Spain), and; CD68-FITC obtained from An der Grub (ADG, Vienna, Austria). For reproducible identification of all nucleated cells and leukocytes present in each sample, staining for the above listed mAb was systematically done in 4-colour stainings in which CD45-PacB and DRAQ5 (Cytognos SL) were combined with the above listed FITC and PE-conjugated mAb reagents. Absence of blood infiltration at single cell suspensions was confirmed based on the lack of CD16^+^CD45^+^ neutrophils in the samples. For cytoplasmic (Cy) markers (Cybcl2 and CyCD68), cells were permeabilized (1 h at −20°C) prior to intracellular staining, as described elsewhere [16]. For further lymphocyte subset analysis, an additional 5-color staining – CD45 Pacific Orange (PacO; Invitrogen), CD3-PacB (BD), CD8-FITC (BD), CD19-FITC (BD) and CD56-PE (Cytognos SL) -, was used. For all tubes, staining with DRAQ5 was performed 5 min prior to the measurement in the flow cytometer, as previously described [17]. To assess control baseline autofluorescence levels, an aliquot of each tumor sample stained only for DRAQ5 was measured in parallel.

Data acquisition was performed for ≥1×10^5^ cells per antibody combination in a FACSCanto II flow cytometer (BD), using the FACSDiva^TM^ 6.0 software (BDB). The INFINICYT software program (Cytognos SL) was used for data analysis, aimed at determining both the percentage of positive cells and the amount of protein expressed per cell – mean fluorescence intensity (MFI) – for each individual marker analyzed within each cell population of interest.

### Interphase Fluorescence in situ hybridization (iFISH)

For all freshly-frozen tumor samples obtained during surgery, iFISH analysis was performed for a panel of 12 probes specific for chromosomes 1p36/1q25, CEP 7/10, 9p34/22q11.2 (LSI BCR/ABL), 14q32.3/18q21 (LSI IgH/BCL2), 15q22/17q21 (LSI PML/RAR-α) and CEP X/Y (Vysis Inc., Downers Grove, IL) according to previously described double-staining techniques [7].

### Gene expression profiling (GEP)

In a subset of 40 meningioma samples (28 WHO grade I, 11 WHO grade II and 1 WHO grade III tumors), GEP was analyzed with the Human Genome 133A Affymetrix array (Affymetrix Inc, Santa Clara, CA, USA). After thawing, tumors were homogenized with a Potter-'S'-Elvehjem homogenizer (Uniform, Jencons, UK) and total RNA was isolated using TRIzol (Invitrogen) and the RNeasy Mini Kit (QIAGEN, Valencia, CA, USA). The integrity/purity of the purified RNA was determined using a microfluidic electrophoretic system (Agilent 2100 Bioanalyzer; Agilent Technologies, Palo Alto, CA, USA). Then, GEPs were analyzed according to the manufacturer's instructions, using the one-cycle cDNA synthesis kit and the Poly-A RNA gene chip control kit (Affymetrix Inc.), as reported elsewhere [6], [18]. Data files containing data about the expression levels for the 40 tumors were normalized – Robust microarray normalization (RMA) – and analyzed using the R (version 2.7.0; http://www.r-project.org) and Bioconductor (http://www.bioconductor.org) software tools. The microarray dataset is available in the Gene Expression Omnibus (GEO) public data repository (GSE43290 access code). Differentially expressed genes between samples from the different cytogenetic subgroups of meningiomas were identified using a supervised two-class unpaired Significance Analysis of Microarray (SAM) [19], based on a combined cutoff with a false discovery rate of <0.05 (T-test). Further investigation of the altered pathways was performed using the Ingenuity Pathway Analysis (IPA) software (Ingenuity Systems Inc., Redwood City, CA, USA). Through the IPA software the specific cell functions associated with those genes under- or over-expressed in each specific cytogenetic subgroup of meningiomas were first investigated. In a second step, the IPA software was used for a more detailed analysis of those signaling pathways involving genes which were under- and/or over-expressed among meningiomas with isolated monosomy 22/del(22q) vs other meningioma tumors. For this purpose, those genes being associated with the highest scored ‘Bio Functions’ for this specific cytogenetic subgroup of meningiomas were selected (e.g. cell growth and proliferation of immune cells; hematological system development and function; immune cell trafficking; cell-to-cell signaling and interaction of immune cells; inflammatory response). In the following step, those genes showing more ‘Direct Relationships’ as well as those associated with ‘Antigen Presenting Cell functions’ were selected for the ‘Path Designer’ tool of the IPA software.

### Immunohistochemical analyses

In a representative subgroup of samples (n = 12), immunostaining of macrophages was performed with the anti-CD68 antibody to confirm tissue localization of these cells. For this purpose, 3µm-thick tissue sections were cut from paraffin-embedded blocks, deparaffinized, and stained in a Leica-BOND-III automated immunostainer (Leica Biosystems, Wetzlar, Germany) using the Bond Polymer Refine Detection kit (Leica Biosystems), according to the manufacturer's instructions. Briefly, after rehydrated, antigen retrieval was achieved with citrate buffer (pH 6.0) and endogenous peroxidase was blocked by incubation in 3% hydrogen peroxide. Sections were then incubated with an anti-CD68 mAb (clone KP1, dilution 1∶50; Master Diagnóstica, Granada, Spain), for 20 minutes, followed by the rabbit anti-mouse Post Primary, the polymeric horseradish peroxidase (HRP) and its 3,3′-diaminobenzidine (DAB) substrate as the final chromogen. All immunostained sections were lightly counterstained with hematoxylin. Analysis was performed in an Olympus BX5 microscope equipped with a 100x oil objective (Olympus, Melville, NY, USA).

### Statistical methods and hierarchical clustering

For each continuous variable analyzed, its median, mean and standard deviation (SD) values, as well as range and both the 25^th^ and 75^th^ and the 10^th^ and 90^th^ percentiles, were calculated; for categorical variables, frequencies were reported. Statistical significance was determined through the non-parametric Kruskal-Wallis and Mann-Whitney U tests (for continuous variables) or the Pearsońs Chi-square test (for categorical variables); the Spearman's correlation was used to explore the degree of correlation between different variables (SPSS 15.0 software, SPSS, Chicago, IL, USA). The Kaplan-Meier method was used to construct relapse-free survival (RFS) curves, and the log-rank test was applied to compare RFS curves. P-values<0.05 (with a FDR correction for multiple comparisons of <10%), were considered to be associated with statistical significance.

The most discriminant cut-off value for low vs high tumor infiltration by inflammatory cells was calculated using receiver operating characteristic (ROC) curve analysis.

For unsupervised clustering analyses, normalization of the datasets was performed for each parameter by calculating the ratio between the value obtained for each sample and the median of all samples analyzed. A logarithmic (base 2) transformation was applied to the ratio values, and the log2 ratios were then used for hierarchical clustering analyses (Cluster 3.0 and Tree View software; Stanford University, Stanford, CA, USA). Unsupervised hierarchical cluster analyses were performed using the Pearson correlation and the average linkage clustering method. Principal component (PC) analysis (PCA) was performed using the MultiExperiment Viewer Software (MeV v4.8, TM4 Microarray Software Suite).

## Results

### Inflammatory infiltrates in meningioma samples and its association with disease features

**Figure 1:**
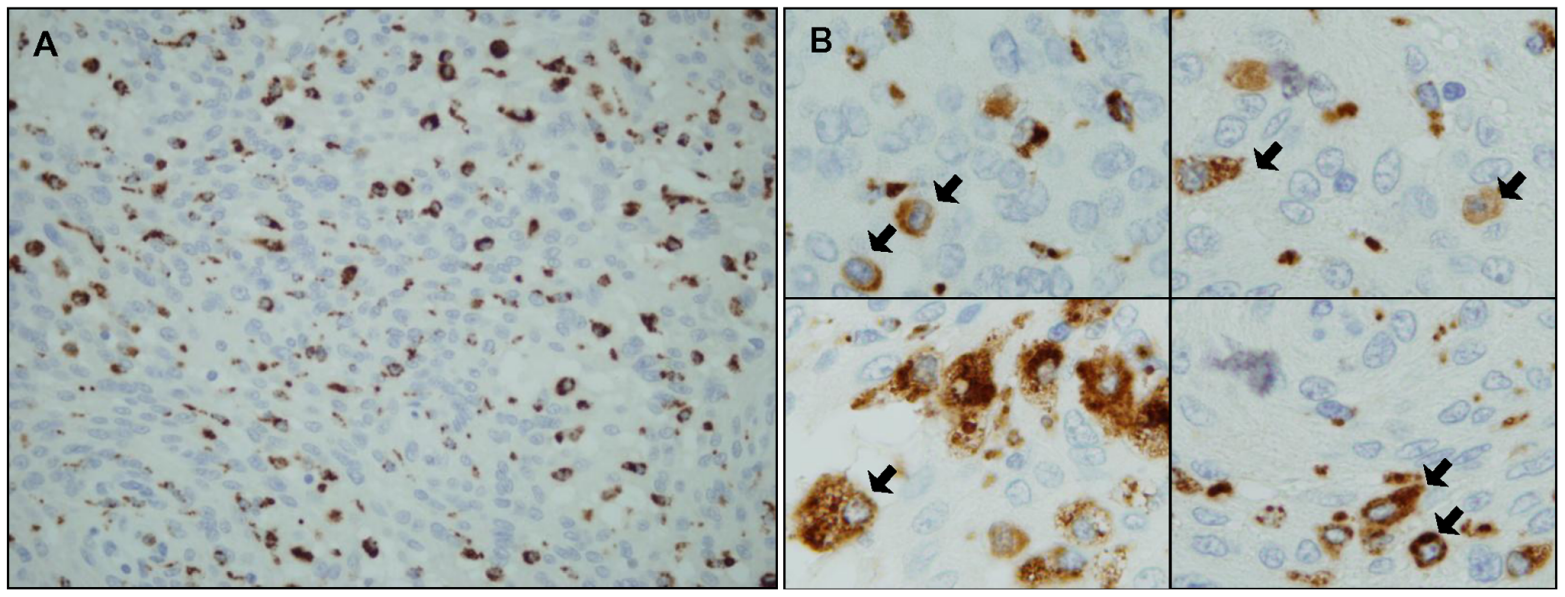
Immunohistochemical staining of meningioma tissues with the anti-CD68 antibody. CD68-positive cells detected within the tumor parenchyma showed reactivity in their cytoplasm and a mononuclear cell appearance, compatible with macrophages infiltrating the tumor. An overview of the whole tissue from a representative case (original magnification, ×400) (panel A) and a higher amplification of areas containing CD68-positive cells (original magnification, ×1000) (panel B), are displayed.

As previously described [10], all meningioma samples showed infiltration by inflammatory and other immune cells by flow cytometry, although their percentage was highly variable among the distinct tumors. In order to investigate the potential association between the amount of the inflammatory infiltrate by flow cytometry and other features of the disease, patients were divided into cases with low (<23%) and high (≥23%) percentage of the most represented inflammatory cells (CD14^+^ HLA-DR^+^ CD45^+^ TiMa) in the tumor infiltrates, based on ROC curve analysis – area under the curve (AUC) of 90% (p<0.001) for the selected cutoff (23% of TiMa) -. Immunohistochemical expression of CD68 was detected in the cytoplasm of morphologically heterogeneous mononuclear cells scattered within the tumor tissue, as single cells or groups of cells, only occasionally localized in perivascular areas (Figure 1). On the basis of their immunophenotype, morphology and localization, these cells were identified as mainly corresponding to macrophages infiltrating the tumor.

Comparison of patients with low (<23% TiMa) vs high (≥23% TiMa) levels of tumor infiltration by inflammatory cells (Table 1) showed a clearly different distribution of tumors according to their iFISH cytogenetic profiles (p = 0.001); conversely, both groups of patients showed a similar distribution according to age, sex, tumor localization, histopathological subtypes and WHO tumor grade, together with a similar degree of brain edema and frequency of relapses (p>0.05). From the cytogenetic point of view, a highly significant association was found between meningiomas carrying isolated monosomy 22/del(22q) alone and high levels of infiltration by TiMa, most tumors carrying isolated monosomy 22/del(22q) (14/17 cases, 82%) showing infiltration by ≥23% TiMa (Table 1).

**Table 1:** Clinical, biological and cytogenetic characteristics of meningioma patients with high (≥23%) versus low (<23%) degree of tumor infiltration by tissue macrophages (TiMa; n = 51).

		Total cases (n = 51)	% of TiMa <23 (n = 29)	% of TiMa ≥23 (n = 22)	*p-value*
**Age (years)**		58±13	59±14	56±12	NS
**Gender**	Female	36 (71%)	19 (66%)	17 (77%)	NS
	Male	15 (29%)	10 (44%)	5 (23%)	
**Tumor localization**	Convexity	17 (33%)	7 (24%)	10 (45%)	NS
	Cranial base	14 (27%)	11 (38%)	3 (14%)	
	Convexity/Parasagittal	8 (16%)	4 (13%)	4 (18%)	
	Parasagittal	6 (12%)	3 (10%)	3 (14%)	
	Tentorial	2 (4%)	2 (7%)	0 (0%)	
	Intraosseous	1 (2%)	1 (4%)	0 (0%)	
	Spinal	3 (6%)	1 (4%)	2 (9%)	
**Tumor grade**	Grade I	46 (90%)	25 (86%)	21 (95%)	NS
	Grade II	3 (6%)	2 (7%)	1 (5%)	
	Grade III	2 (4%)	2 (7%)	0 (0%)	
**Tumor histopathology**	Meningothelial	14 (27%)	9 (31%)	5 (23%)	NS
	Transitional	16 (31%)	10 (35%)	6 (27%)	
	Psammomatous	7 (14%)	3 (10%)	4 (18%)	
	Fibroblastic	7 (14%)	1 (3%)	6 (27%)	
	Other *	7 (14%)	6 (21%)	1 (5%)	
**iFISH karyotype**	Diploid	17 (33%)	14 (48%)	3 (14%)	0.001
	Monosomy 22/del(22q)	17 (33%)	3 (10%)	14 (63%)	
	Complex	16 (32%)	11 (38%)	5 (23%)	
	del(1p)	1 (2%)	1 (4%)	0 (0%)	
**Edema**	No/light	30 (59%)	14 (48%)	16 (73%)	NS
	Moderate/severe	21 (41%)	15 (52%)	6 (27%)	
**Relapses**	Yes	4 (8%)	1 (4%)	3 (14%)	NS
	No	47 (92%)	28 (96%)	19 (86%)	

NS, statistically no significant differences observed (*p>*0.05).

* Includes one angiomatous, one secretory, one rhabdoid, one papillary and three atypical meningioma cases.

Of note, whereas cases with either a diploid karyotype or isolated monosomy 22/del(22q) tumors showed a longer RFS than meningioma patients carrying complex karyotypes (p = 0.01), the level of tumor infiltration by TiMa on itself did not show a significant impact on patient outcome (p>0.05).

### The immunophenotypic profile of inflammatory cells in tumor infiltrates varies according to tumor cytogenetics

Based on the iFISH analyses for chromosomes 1p36/1q25, 7, 9p34, 10, 14q32.3, 15q22, 17q21, 18q21, 22q11.2, X and Y, patients were classified according to tumor cytogenetics as having a diploid karyotype – 29/75 cases (39%) -, isolated monosomy 22/del(22q) – 26/75 cases (35%) – and a complex iFISH karyotype with losses and/or gains of two or more chromosomes – 19/75 cases (25%); the remaining case showed an isolated loss of chromosome 1p.

**Figure 2:**
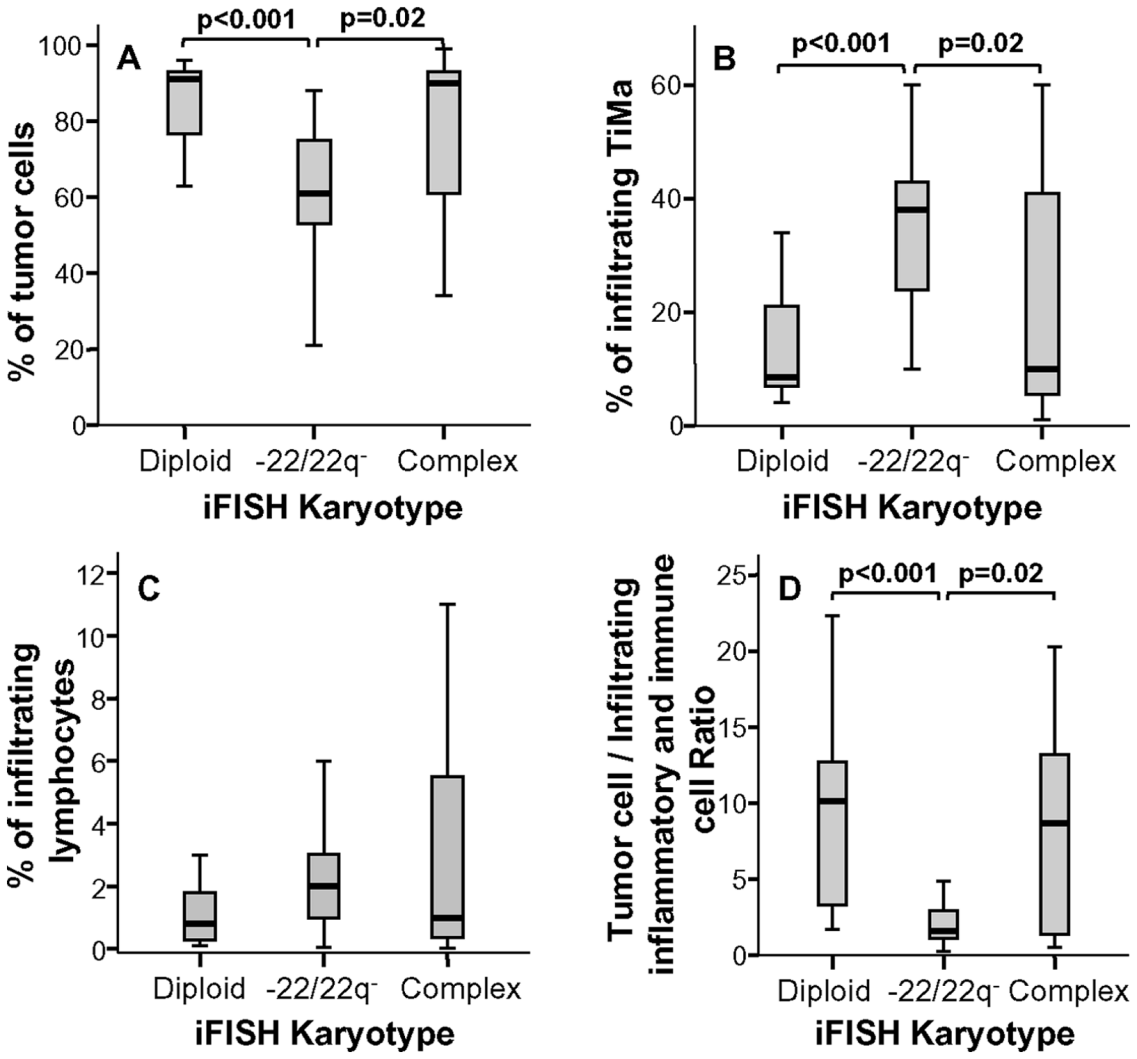
Distribution of tumor cells, inflammatory and other immune cells in meningioma samples classified according to the cytogenetic profile of the tumor. Percentage of tumor cells (panel A), tissue macrophages (TiMa) (panel B) and total lymphocytes infiltrating meningioma samples (panel C), grouped according to the cytogenetic iFISH profile of the tumor, are shown, as also the ratio between the number of tumor cells and all other infiltrating cells (panel D). Notched-boxes represent 25^th^ and 75^th^ percentile values; the lines in the middle and vertical lines correspond to median values and the 10^th^ and 90^th^ percentiles, respectively.

**Figure 3:**
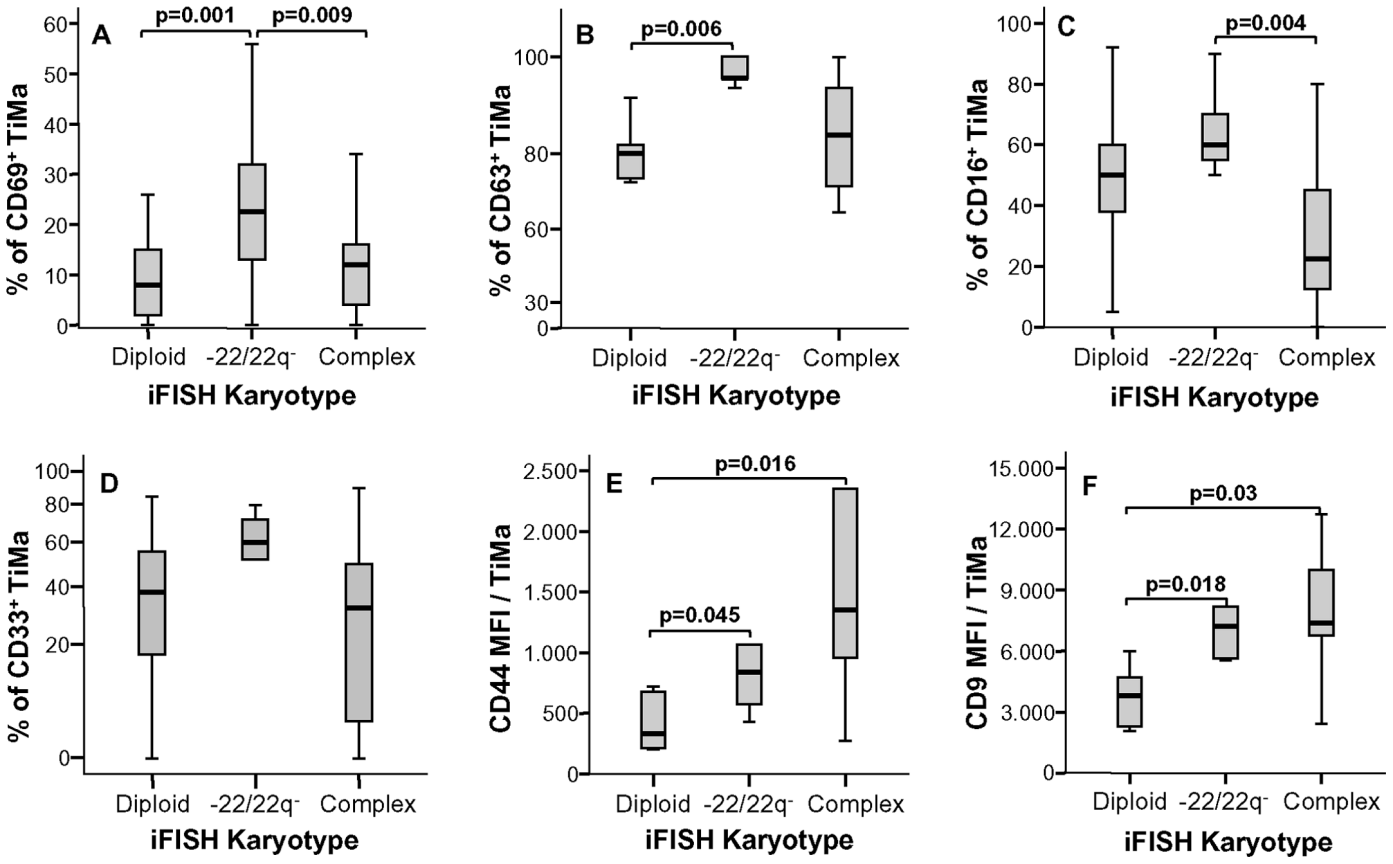
Immunophenotype of Tissue macrophages (TiMa) infiltrating meningioma samples, according to the cytogenetic profile of tumor cells. The percentage of TiMa expressing CD69 (panel A), CD63 (panel B), CD16 (panel C) and CD33 (panel D) are shown together with the mean amount of CD44 (panel E) and CD9 (panel F) expressed per TiMa infiltrating meningioma samples, according to the iFISH profile of tumor cells. MFI, mean fluorescence intensity (arbitrary fluorescence units) per cell. Notched-boxes represent 25^th^ and 75^th^ percentile values; the lines in the middle and vertical lines correspond to median values and the 10^th^ and 90^th^ percentiles, respectively.

A more detailed analysis of the immunophenotypic features of TiMa from meningioma samples with distinct karyotypes showed that cases with isolated monosomy 22/del(22q) not only displayed significant increased levels of infiltration by TiMa versus meningiomas with both diploid (p<0.001, Figure 2B) and complex karyotypes (p = 0.02, Figure 2B), but they also showed a distinct immunophenotypic profile for such TiMa (Figure 3). Accordingly, TiMa from meningiomas with isolated monosomy 22/del(22q) showed increased expression levels of several activation-associated markers with higher percentages of CD69^+^ (p≤0.009 vs diploid and complex tumors; Figure 3A) and CD63^+^ TiMa (p = 0.006 vs diploid cases; Figure 3B). In addition, TiMa from meningiomas with isolated monosomy 22/del(22q) also displayed a higher percentage of CD16^+^ cells vs tumors with complex karyotypes (p = 0.004; Figure 3C); despite not statistically significant, higher percentages of CD33^+^ cells were also associated with −22/22q^−^ cases (Figure 3D). In turn, they showed intermediate levels of expression of both the CD44 and CD9 adhesion molecules, between those of diploid and complex karyotype tumors (p<0.05; Figures 3E and 3F).

**Figure 4:**
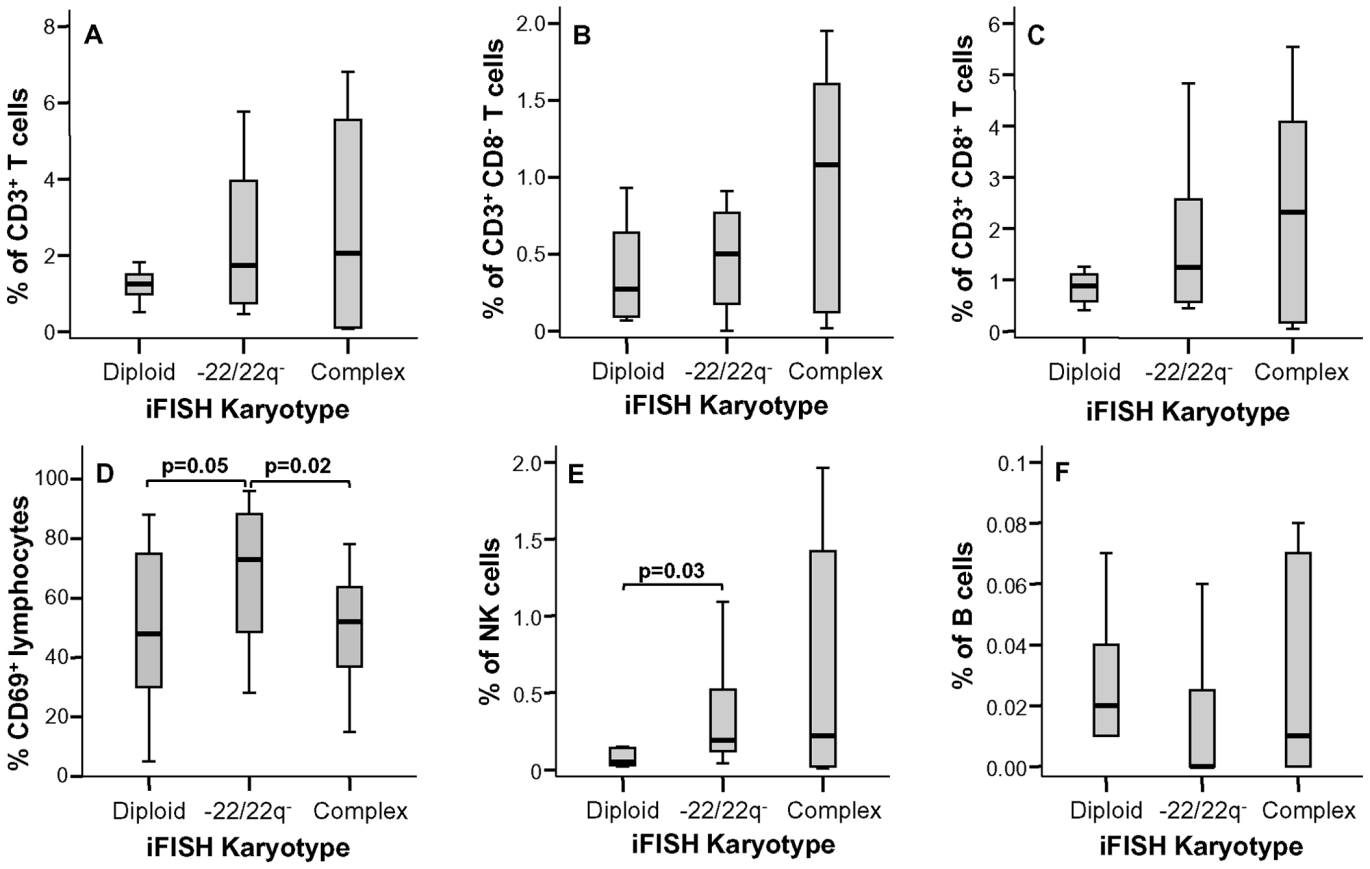
Distribution of the major lymphocyte subsets and activated CD69^+^ lymphocytes in inflammatory infiltrates of meningiomas classified according to the cytogenetic profile of tumor cells. The percentage of total CD3^+^ T cells (panel A), CD3^+^ CD8^−^ T cells (panel B), CD3^+^ CD8^+^ T cells (panel C), activated CD69^+^ lymphocytes (panel D), CD3^−^ CD19^−^ 56^+^ NK cells (panel E) and CD3^−^ CD19^+^ B cells (panel F) are shown. Notched-boxes represent 25^th^ and 75^th^ percentile values; the lines in the middle and vertical lines correspond to median values and the 10^th^ and 90^th^ percentiles, respectively.

Conversely, no significant differences (p>0.05) were found regarding the amount of lymphocytes infiltrating the tumor (Figure 2C), neither their major subsets (Figure 4), except for higher numbers of NK-cells in cases with isolated monosomy 22/del(22q) vs diploid tumors (p = 0.03; Figure 4E). Interestingly, this was also associated with an increased percentage of CD69^+^ lymphocytes among meningiomas with isolated monosomy 22/del(22q) vs both cases with diploid and complex iFISH karyotypes (p<0.05; Figure 4D).

**Figure 5:**
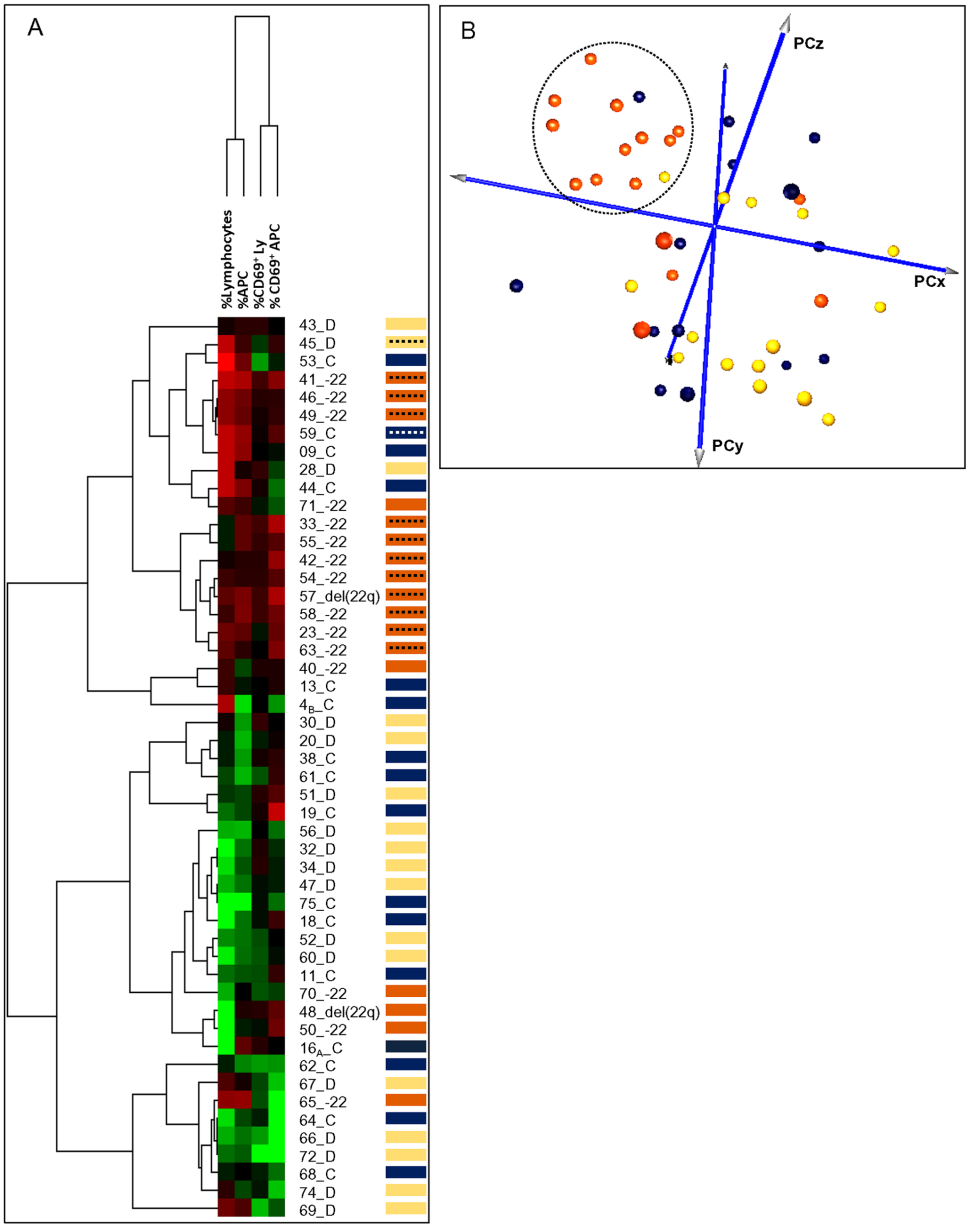
Hierarchical clustering analysis of meningioma samples based on the relative distribution and the activation-associated (CD69^+^) immunophenotypic profile of infiltrating inflammatory cells and lymphocytes: relationship with the cytogenetic subgroups of the disease. Results are presented in a matrix format where each column represents a single immunophenotypic variable and each row corresponds to a different meningioma sample (rows annotated as ‘D’, ‘−22’ and ‘C’ correspond to meningiomas with diploid, isolated monosomy 22/del(22q) and complex iFISH karyotypes, respectively). Normalized values are represented by a color scale where red and green colors reflect values above and below the mean values obtained for each variable, respectively (panel A). A 3-dimensional principal component analysis (PCA) representation of all meningioma samples based on the number and features of inflammatory cells and lymphocytes infiltrating the tumor, as analyzed by flow cytometry (n = 51) is displayed; as shown there, most tumors with isolated monosomy 22/del(22q) (orange dots) tend to cluster together based on the pattern of infiltration of the tumor by inflammatory and other immune cells (panel B).

In line with the above observations, unsupervised hierarchical clustering analysis, based on the number and immunophenotypic features of the inflammatory tumor infiltrates of each meningioma, showed two major clusters of tumors (Figure 5A): one group (group A) included the great majority of patients (13/17, 76%) with meningiomas carrying isolated monosomy 22/del(22q), whereas the other group (group B) comprised most patients with a diploid (14/17, 82%) or complex karyotype (10/16, 63%). As expected, group A cases were characterized by both higher levels of infiltration by TiMa and lymphocytes, and greater percentages of CD69^+^ activated TiMa and lymphocytes. Additionally, principal component analysis based on the percentages of lymphocytes and TiMa infiltrating meningioma samples and their immunophenotypic features, also identified a homogeneous group of samples (n = 13/51) which included almost only cases with isolated monosomy 22/del(22q) (11/13, 85%; Figure 5B).

### Association between the gene expression profile and iFISH karyotype of meningiomas and the pattern of infiltration of the tumor by inflammatory and other immune cells

**Figure 6:**
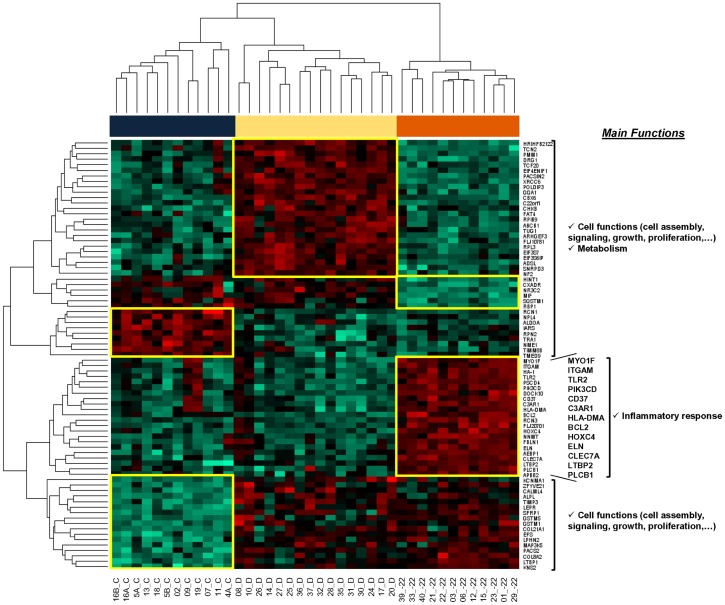
Hierarchical clustering analysis of the GEP of meningioma samples. Results are presented in a GEP matrix format where each row represents a single gene (listed with the corresponding gene symbol) and each column corresponds to a distinct meningioma sample (n = 40); those columns identified as ‘D’ (colored yellow), ‘−22’ (colored orange) and ‘C’ (blue color) correspond to individual meningioma tumors with a diploid, monosomy 22/del(22q) and complex iFISH karyotype, respectively. Normalized values are represented by a color scale where red and green colors indicate values above and below the mean mRNA expression values, respectively. Hierarchical clustering analysis was based on the expression of those 79 genes which showed the highest classification power for the three cytogenetic subgroups of meningiomas. On the right side of the plot, the major common functions of the listed genes, based on the analyses performed with the Ingenuity Pathway software, are indicated. As displayed, genes overexpressed in meningiomas carrying monosomy 22/del(22q) are mainly involved in inflammatory cell functions.

In order to better understand the molecular mechanisms that could contribute to explain the association observed between the pattern of infiltration of meningioma samples by inflammatory cells and the tumor iFISH karyotype, we further investigated the GEP of 40 meningioma samples using DNA oligonucleotide arrays. Unsupervised hierarchical clustering analysis using those 79 genes, for which the highest variation among tumors with distinct iFISH cytogenetic patterns was observed, showed clear separation among meningioma samples displaying distinct karyotypes, according to their mRNA expression profiles (Figure 6). Analysis of the functional role of these 79 genes associated with the different cytogenetic groups of meningiomas, showed that cases with isolated monosomy 22/del(22q) were specifically characterized by an increased expression of a set of genes which are related to the inflammatory response and to signaling/activation of immune cells. Among other genes, these included the *BCL2*, *C3AR1*, *CD37*, *CLEC7A*, *ELN*, *HLA*-*DMA*, *HOXC4*, *ITGAM*, *LTBP2*, *MYO1F*, *PIK3CD*, *PLCB1* and *TLR2* genes (Figure 6). Conversely, diploid tumors were mainly characterized by overexpression of a group of genes, (e.g. *ABCB1*, *ADSL*, *CHKB*, *PACSIN2*, *PMM1* and *TCN2* genes) which are mainly involved in small molecule metabolism and cellular biochemistry, including also the *NF2* gene. Finally, tumors with complex karyotypes were characterized by a greater expression of the *ALDOA*, *TRA1*, *NME1*, *NPLOC4* and *TMED9* genes, as well as by decreased levels of the *ALPL*, *COL8A2*, *EFS*, *GSTM1*, *GSTM5*, *KCNMA1*, *KNS2*, *LEPR*, *LPHN2*, *LTBP1*, *MAP3K5*, *PACS2*, *SFRP1*, *TIMP3* and *ZFYVE21* genes, most of such genes being mainly involved in cellular functions related to cell death, cell cycle, cell growth and proliferation, and to cellular assembly.

**Figure 7:**
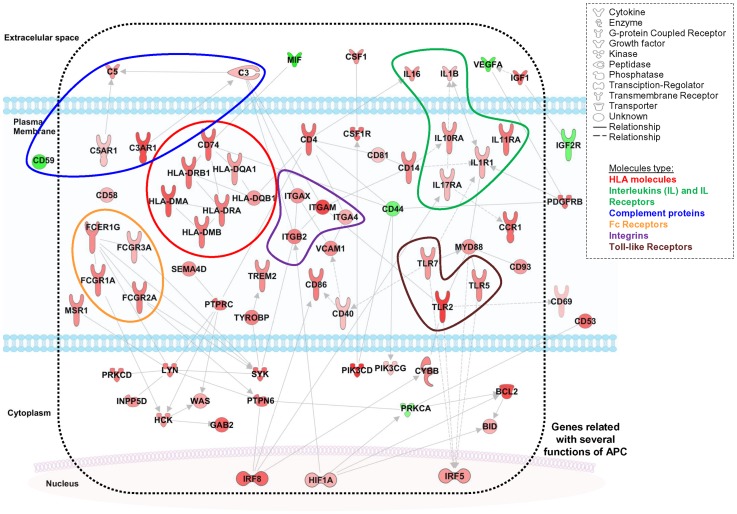
Schematic representation of the functional impact of GEP of meningiomas with isolated monosomy 22/del(22q). The scheme was built based on the results obtained through the analysis of GEP performed with the Ingenuity Pathway Analysis software and it shows increased expression of several inflammatory genes, particularly genes involved in antigen presenting cell functions, among cases with isolated monosomy 22/del(22q). Such genes include HLA and HLA-associated molecules (*HLA-DMA*, *HLA-DMB*, *HLA-DRA*, *HLA-DRB1*, *HLA-DQA1*, *HLA-DQB* and *CD74*), cytokines (*IL16*, *IL1B*, *IL1R1*, *IL10RA*, *IL11RA* and *IL17RA*), growth factors and growth factor receptors (*CSF1*, *CSF1R*, *IGF1*, *IGF2R*, *VEGF* and *PDGFRB*), complement proteins (*C5*, *C3*, *C3AR1*, *C5AR1* and *CD59*), immunoglobulin Fc (FcIg) receptors (*FCGR1A*, *FCGR2A*, *FCGR3B* and *FCER1G*) and the *CCR1* chemokine receptor, integrins (*ITGAM*, *ITGAX*, *ITGA4* and *ITGB2*) and other adhesion molecules (*VCAM1*, *CD44*, *CD53*, *CD58*, *CD81* and *CD93*), immune co-stimulatory molecules (*CD4*, *CD40* and *CD86*), toll-like receptors (*TLR2*, *TLR5* and *TLR7*) and TLR-associated molecules (*CD14* and *MYD88*), together with phosphoinositide-3-kinases (*PIK3CG* and *PIK3CD*) and other kinases (*PRKCA*, *PRKCD*, *SYK*, *LYN* and *HCK*), tyrosine phosphatases (*PTPRC* and *PTPN6*), and apoptotic proteins (*BCL2* and *BID*), together with other signaling molecules (*CD69*, *CYBB*, *GAB2*, *HIF1A*, *INPP5D*, *IRF5*, *IRF8*, *MIF*, *MSR1*, *SEMA4D*, *TREM2*, *TYROBP* and *WAS*).

A more detailed functional analysis of the specific inflammatory pathways involved in meningiomas with isolated monosomy 22/del(22q) (IPA software) showed involvement of inflammatory response genes which are specifically associated with immune responses, cell adhesion, motility and activation and recruitment of antigen presenting cells and/or macrophages (Figure 7). Altered genes included HLA and HLA-associated molecules (*HLA*-*DMA*, *HLA*-DMB, *HLA-DRA*, *HLA-DRB1*, *HLA-DQA1*, *HLA-DQB* and *CD74*), inflammatory cytokines (*IL16*, *IL1B*, *IL1R1*, *IL10RA*, *IL11RA* and *IL17RA*), complement proteins (*C5*, *C3*, *C3AR1* and *C5AR1*), immunoglobulin Fc (FcIg) receptors (*FCGR1A*, *FCGR2A*, *FCGR3B* and *FCER1G*) and the *CCR1* chemokine receptor, integrins (*ITGAM*, *ITGAX*, *ITGA4* and *ITGB2*) and other adhesion molecules (*VCAM1*, *CD53*, *CD58*, *CD81* and *CD93*), immune co-stimulatory molecules (*CD4*, *CD40* and *CD86*), toll-like receptors (*TLR2*, *TLR5* and *TLR7*) and TLR-associated molecules (*CD14* and *MYD88*), growth factors and growth factor receptors (*CSF1*, *CSF1R* and *IGF1*), apoptosis-associated proteins (*BCL2* and *BID*), together with phosphoinositide-3-kinases (*PIK3CG* and *PIK3CD*) and other kinases (*PRKCD*, *SYK*, *LYN* and *HCK*), tyrosine phosphatases (*PTPRC* and *PTPN6*), and signaling molecules (*CD69*, *CYBB*, *GAB2*, *HIF1A*, *INPP5D*, *IRF8*, *MSR1*, *SEMA4D*, *TREM2*, *TYROBP* and *WAS*) (Figure 7).

Noteworthy, a significant correlation was observed in those 13 meningioma samples in which GEP and flow cytometry immunophenotyping were performed in parallel, for the percentage of inflammatory and other immune cells infiltrating the tumor and the mRNA levels of proteins specifically expressed by these cells (e.g. TiMa) such as HLA-DR (r^2^ = 0.8; p<0.001), CD14 (r^2^ = 0.8; p<0.001), Cybcl2 (r^2^ = 0.7; p = 0.01), CD53 (r^2^ = 0.7; p = 0.01), CD37 (r^2^ = 0.7; p = 0.01), CD99 (r^2^ = 0.6; p = 0.02), CD45 (r^2^ = 0.6; p = 0.03), CD16 (r^2^ = 0.6; p = 0.04), CyD68 (r^2^ = 0.6; p = 0.04), and HLA-I (r^2^ = 0.6; p = 0.04).

## Discussion

According to the World Health Organization, meningiomas are mostly classified as grade I benign tumors [2]; however, grade I meningiomas are genetically very heterogeneous [6]. Accordingly, distinct cytogenetic profiles have been identified in meningiomas, which include (i) diploid tumors, (ii) tumors showing isolated monosomy 22/del(22q), (iii) del(1p36) alone, (iii) isolated loss of a sex chromosome, and (iv) meningiomas with complex karyotypes in the absence or (v) presence of del(1p36) and/or monosomy 14. From the prognostic point of view, meningiomas which have complex karyotypes, particularly those carrying del(1p36) and/or monosomy 14, display a significantly worse outcome, whereas diploid tumors and cases with isolated monosomy 22/del(22q) have a particularly good prognosis [6], as confirmed also in our series.

At present, the specific factors that contribute to the better outcome of −22/22q^−^ cases remain to be elucidated. Previous studies have claimed that monosomy 22/del(22q) is frequently associated with NF2 mutation, the later potentially representing the first chromosomal alteration to occur in meningiomas; if this hypothesis holds true, cases carrying an isolated loss of chromosome 22 could represent the earliest stage of neoplastic transformation in meningiomas [2]. However, more recent studies in which the intratumoral patterns of cytogenetic evolution have been analyzed in detail indicate that this is probably not the case; more likely, loss of chromosome 22/*NF2* mutation representing one of multiple pathways of intratumoral clonal evolution occurring in benign grade I meningiomas [7]. In line with this hypothesis, Clark et al. have recently reported distinct genome profiles of meningiomas based on the presence versus absence of *NF2* mutations, non-*NF2* mutated meningiomas frequently showing mutations in other genes (e.g. *TRAF7*, *KLF4*, *AKT1* and *SMO*) [20].

In turn, it should be taken into account that tumor behavior depends not only on tumor cytogenetics, but also on the tumor microenvironment, including surrounding cells which may either support tumor growth or control the disease [21]. In this regard, the potential role of immune cells infiltrating the tumor has become particularly relevant, as the presence of inflammatory and both cytotoxic and regulatory cells has been correlated with the behavior of the disease (e.g. patient outcome) in multiple different tumor types [11]. In the present study, we show a clear association between the levels of immune/inflammatory cells infiltrating meningiomas and tumor cytogenetics. Tumor infiltration by immune/inflammatory cells had already been shown to be associated in meningiomas with both tumor grade and the histopathological subtypes [9], [13]. By contrast, to the best of our knowledge, this is the first report which shows a clear relationship between inflammatory/immune infiltrates and tumor cytogenetics in meningiomas. Overall, meningiomas carrying isolated monosomy 22/del(22q) showed significantly greater numbers of TiMa infiltrating the tumor, together with a more pronounced activation profile of immune cells, as reflected by greater percentages of CD69^+^ and CD63^+^ TiMa and/or lymphocytes [22], [23], versus cases with either a diploid or a complex iFISH karyotype. In addition, HLA-DR^+^CD14^+^CD45^+^CD68^+^ TiMa from patients with isolated monosomy 22/del(22q) also showed higher levels of expression of CD16, an FcγRIII receptor typically absent in recently produced blood monocytes, but expressed during macrophage maturation in peripheral tissues (e.g. alveolar and pleural macrophages) [24], [25]. From a functional point of view, expression of CD16 enables macrophages to carry out antibody-dependent cell-mediated cytotoxic functions, which would make them more efficient phagocytic cells [26]. Altogether, these results suggest that in addition to the greater numbers of TiMa infiltrating the tumor, TiMa from meningiomas carrying monosomy 22/del(22q) alone would also show a more activated and functionally matured phenotype. In this regard, TiMa of both −22/22q^−^ and cytogenetically complex meningiomas also showed higher expression of both the CD44 and CD9 adhesion molecules vs diploid cases. CD44 is a cell-adhesion molecule which is expressed by macrophages [27], and has been previously reported to be up-regulated in tumor-associated macrophages, playing a role in their recruitment and activation [28], [29]. Similarly, the CD9 tetraspanin has also been reported to promote activation of macrophages through its functional association with Fcγ receptors [30]. These observations further support a central role for TiMa in controlling tumor growth, as well as in promoting homing/chemoattraction of inflammatory and other immune cells to the tumor, among meningiomas with isolated monosomy 22/del(22q), which could potentially contribute to explain the better outcome of this specific subgroup of meningioma patients vs cases with complex karyotypes.

In line with this hypothesis, meningiomas with isolated monosomy 22/del(22q) also displayed a greater infiltration by NK cells and lymphocytes expressing the CD69 early-activation antigen. Although NK cells and CD69^+^ activated lymphocytes only represented a small fraction of all infiltrating cells they may also contribute to immune surveillance and to the elimination of tumor cells and thus, to control tumor growth through direct cytotoxic mechanisms cooperating with those of tissue macrophages [31].

To further investigate the molecular mechanisms involved in inflammatory and immune responses in those tumors carrying isolated monosomy 22/del(22q) vs other meningiomas, we further analyzed the GEP of tumor samples from the distinct cytogenetic subgroups of meningiomas. As expected, meningiomas with isolated monosomy 22/del(22q) typically showed a GEP associated with an increased inflammatory and immune response consisting of greater expression of genes involved in antigen presentation (e.g. HLA and HLA-associated molecules), phagocytosis (Fc receptors) and cell activation/cell signaling (e.g. immune co-stimulatory molecules, toll-like receptors and inflammatory cytokines), when compared to tumors with diploid and complex karyotypes.

So far, two distinct populations of functionally polarized macrophages have been described, which are generated depending on the cytokines present in the tissue microenvironment: classical M1 macrophage which develop under the influence of LPS and IFN-γ, produce pro-inflammatory cytokines (e.g. IL-12, IL-1, and IL-6), mediate resistance to pathogens and contribute to tissue destruction, and; M2 macrophages, developed under the influence of IL-4 and IL-10, which produce anti-inflammatory cytokines (e.g. IL-10 and TGF-β), promote tissue repair and remodeling and support to tumor progression [32]. Despite lacking specific markers, M1 macrophages express receptors like CD16, CD32, CD64 and CD86, while M2 macrophages are characterized by abundant levels of CD163 and CD206. Although until now there is no information about the type of macrophages that infiltrate meningiomas, the higher expression levels of CD16 (FCGR3A) found here both at the protein and mRNA levels, together with the increased mRNA levels of *CD86*, *CD32* (*FCGR2A*) and *CD64* (*FCGR1A*) observed in meningiomas with monosomy 22/del(22q) alone, support an M1 vs M2 polarization of macrophages in this subgroup of meningiomas and consequently also, a more favorable anti-tumoral microenvironment. In line with this, NK cells have been reported to play an indirect role in redirecting macrophage activation toward the M1 phenotype [32], [33], NK cells being also found at higher numbers in our series of meningiomas with an isolated −22/22q^−^ karyotype. Similarly, higher levels of expression of *IRF5* and *IRF8*, but not *IRF4*, were reported as part of the GEP characteristic of −22/22q^−^ meningiomas; while *IRF5* production has been shown to play a critical role in M1 macrophage polarization [34], IRF4 stimulates expression of M2 macrophage markers [35]. Altogether these results support a predominant M1 polarization of macrophages in meningiomas with isolated monosomy 22/del(22q) and potentially also their better prognosis versus other cytogenetic subtypes of meningiomas (e.g. cases with complex karyotypes). Further investigations about the functional behavior of infiltrating macrophages in meningiomas are needed to confirm this hypothesis.

Whether or not the inflammatory responses in meningiomas are directly determined by the loss of expression in tumor cells of genes specifically coded in chromosome 22/22q, also deserves further investigation. Despite this, it should be noted that the most significant immune response-associated gene coded in chromosome 22, which was lost in this cytogenetic subgroup of meningiomas, is the *MIF* gene. MIF was originally identified as a T-cell-derived factor responsible for the inhibition of macrophage migration [36]. However, nowadays MIF has been recognized to act as a pro-inflammatory cytokine which is both involved in inflammatory and immune responses, as well as in tumor cell growth and invasiveness [36], [37]. In this regard, recent studies indicate that MIF protein levels are elevated in cancer patients [37], [38] and that MIF expression directly correlates with stage, metastatic spread, disease-free survival and tumor-associated neovascularization in e.g. lung, prostate, breast and gastric cancer, as well as glioma patients [37], [39], [40], [41], [42], [43]. Thus, loss of MIF in meningiomas with isolated monosomy 22/del(22q) may also play an important role in determining the more indolent behavior and the good prognosis of this subgroup of meningioma patients.

In summary, our results indicate that an increased infiltration of the tumor by tissue macrophages, NK cells and activated lymphocytes in meningiomas, is specifically associated with cases carrying an isolate monosomy 22/del(22q). Whether such enhanced inflammatory/immune infiltrates is due to the loss of expression of specific genes coded in chromosome 22 and whether it reflects an increased anti-tumoral response contributing to disease control and the better outcome of these patients, deserves further investigations.

## Supporting Information

Table S1 **Relevant clinical, histopathological, and genetic characteristics of the 78 meningioma samples studied by multiparameter flow cytometry immunophenotyping (n = 38), gene expression profiling by oligonucleotide arrays (n = 27) or both (n = 13).**
(DOC)
