# Supplementary material for: Association between Inflammatory Infiltrates and Isolated Monosomy 22/del(22q) in Meningiomas
Source: PLoS One. 2013 Oct 1;8(10):e74798. doi: 10.1371/journal.pone.0074798 (PMC3788099; doi:10.1371/journal.pone.0074798)
Supplement: Table S1 — Relevant clinical, histopathological, and genetic characteristics of the 78 meningioma samples studied by multiparameter flow cytometry immunophenotyping (n = 38), gene expression profiling by oligonucleotide arrays (n = 27) or both (n = 13). (DOC) [file pone.0074798.s001.doc]

**Table S1.** Relevant clinical, histopathological, and genetic characteristics of the 78 meningioma samples studied by multiparameter flow cytometry immunophenotyping (n=38), gene expression profiling by oligonucleotide arrays (n=27) or both (n=13).

| **Tumor ID** | **iFISH Karyotype** | **Gender** | **Age** | **Histopathologic subtype (WHO)** | **Tumor grade** | **Localization** | **Edema** | **Tumor relapse** | **Analysis** |
| --- | --- | --- | --- | --- | --- | --- | --- | --- | --- |
| 8 | Diploid | F | 62 | Atypical | II | Spinal | No | No | GEP |
| 10 | Diploid | F | 62 | Atypical | II | Spinal | No | No | GEP |
| 14 | Diploid | F | 76 | Transitional | I | Parasagittal | Light (+) | No | GEP |
| 17 | Diploid | F | 73 | Transitional | I | Cranial base | No | No | GEP |
| 20 | Diploid | M | 56 | Transitional | I | Cranial base | No | No | MFC/GEP |
| 24 | Diploid | F | 68 | Transitional | I | Cranial base | No | No | GEP |
| 25 | Diploid | F | 63 | Psammomatous | I | Cranial base | No | No | GEP |
| 26 | Diploid | F | 84 | Secretory | I | Convexity | Severe (+++) | No | GEP |
| 27 | Diploid | F | 36 | Transitional | I | Spinal | No | No | GEP |
| 28 | Diploid | F | 68 | Transitional | I | Cranial base | Severe (+++) | No | MFC/GEP |
| 30 | Diploid | F | 69 | Meningothelial | I | Cranial base | Light (+) | No | MFC/GEP |
| 31 | Diploid | M | 77 | Transitional | I | Cranial base | Moderate (++) | No | GEP |
| 32 | Diploid | M | 54 | Transitional | I | Cranial base | Moderate (++) | No | MFC/GEP |
| 34 | Diploid | F | 42 | Meningothelial | I | Tentorial | No | No | MFC |
| 35 | Diploid | F | 47 | Meningothelial | I | Cranial base | No | No | GEP |
| 36 | Diploid | M | 65 | Atypical | II | Cranial base | No | No | GEP |
| 37 | Diploid | F | 54 | Atypical | II | Cranial base | Moderate (++) | No | GEP |
| 43 | Diploid | F | 42 | Meningothelial | I | Convexity | Moderate (++) | No | MFC |
| 45 | Diploid | F | 43 | Meningothelial | I | Convexity | No | No | MFC |
| 47 | Diploid | F | 42 | Transitional | I | Convexity | Light (+) | No | MFC |
| 51 | Diploid | F | 61 | Secretory | I | Cranial base | Moderate (++) | No | MFC |
| 52 | Diploid | M | 56 | Rhabdoid | III | Convexity/Parasagittal | Severe (+++) | No | MFC |
| 56 | Diploid | F | 63 | Psammomatous | I | Cranial base | Severe (+++) | No | MFC |
| 60 | Diploid | F | 54 | Angiomatous | I | Tentorial | Light (+) | No | MFC |
| 66 | Diploid | F | 61 | Meningothelial | I | Cranial base | No | No | MFC |
| 67 | Diploid | M | 30 | Transitional | I | Parasagittal | No | No | MFC |
| 69 | Diploid | F | 53 | Meningothelial | I | Cranial base | No | No | MFC |
| 72 | Diploid | F | 51 | Meningothelial | I | Cranial base | No | No | MFC |
| 74 | Diploid | F | 69 | Psammomatous | I | Cranial base | Moderate (++) | No | MFC |
| 1 | Monosomy 22 | F | 76 | Fibroblastic | I | Cranial base | No | Yes | GEP |
| 3 | Monosomy 22 | F | 72 | Psammomatous | I | Spinal | No | No | GEP |
| 6 | Monosomy 22 | F | 77 | Transitional | I | Parasagittal | No | No | GEP |
| 12 | Monosomy 22 | F | 59 | Psammomatous | I | Spinal | No | No | GEP |
| 15 | Monosomy 22 | M | 54 | Psammomatous | I | Cranial base | No | No | GEP |
| 21 | Monosomy 22 | F | 81 | Psammomatous | I | Spinal | No | No | GEP |
| 22 | Monosomy 22 | M | 76 | Transitional | I | Spinal | No | No | GEP |
| 23 | Monosomy 22 | F | 54 | Psammomatous | I | Cranial base | No | No | MFC/GEP |
| 29 | Monosomy 22 | F | 74 | Atypical | II | Spinal | No | No | GEP |
| 33 | Monosomy 22 | F | 34 | Fibroblastic | I | Convexity | No | No | MFC/GEP |
| 39 | Monosomy 22 | F | 65 | Psammomatous | I | Spinal | No | No | GEP |
| 40 | Monosomy 22 | F | 53 | Fibroblastic | I | Convexity | Light (+) | No | MFC/GEP |
| 41 | Monosomy 22 | F | 66 | Fibroblastic | I | Convexity | Moderate (++) | No | MFC |
| 42 | Monosomy 22 | F | 49 | Transitional - | I | Convexity | Light (+) | No | MFC |
| 46 | Monosomy 22 | F | 42 | Transitional | I | Convexity/Parasagittal | No | No | MFC |
| 48 | del(22q) | F | 58 | Fibroblastic | I | Convexity/Parasagittal | Severe (+++) | No | MFC |
| 49 | Monosomy 22 | F | 75 | Psammomatous | I | Spinal | No | No | MFC |
| 50 | Monosomy 22 | F | 78 | Psammomatous | I | Spinal | No | No | MFC |
| 54 | Monosomy 22 | F | 56 | Fibroblastic | I | Convexity/Parasagittal | Light (+) | No | MFC |
| 55 | Monosomy 22 | F | 57 | Fibroblastic | I | Cranial base | No | No | MFC |
| 57 | del(22q) | M | 58 | Transitional | I | Parasagittal | Light (+) | Yes | MFC |
| 58 | Monosomy 22 | F | 66 | Psammomatous | I | Convexity | No | No | MFC |
| 63 | Monosomy 22 | F | 69 | Psammomatous | I | Spinal | No | No | MFC |
| 65 | Monosomy 22 | F | 66 | Transitional | I | Parasagittal | No | No | MFC |
| 70 | Monosomy 22 | M | 77 | Meningothelial | I | Cranial base | No | No | MFC |
| 71 | Monosomy 22 | F | 48 | Fibroblastic | I | Parasagittal | No | No | MFC |
| 2 | -(1p/11/18/22) +(1q/7/17) | F | 64 | Atypical | II | Convexity | Moderate (++) | No | GEP |
| 4A | -14 +(1q/22/X) | F | 30 | Anaplastic | III | Convexity | Moderate (++) | Yes | GEP |
| 4B | -14 +(1q/22/X) | F | 30 | Papillary | III | Convexity | Moderate (++) | Yes | MFC |
| 5A | -(1p/22) | M | 73 | Meningothelial | I | Cranial base | Light (+) | Yes | GEP |
| 5B | -(1p/22) | M | 75 | Transitional | I | Cranial base | Light (+) | Yes | GEP |
| 7 | -(1p/14/22/Y) +(15/18) | M | 23 | Atypical | II | Parasagittal | Severe (+++) | No | GEP |
| 9 | -(1p/6/14/22) | M | 41 | Atypical | II | Convexity | Light (+) | Yes | MFC/GEP |
| 11 | -14 +(7/10/15/17) +18 | F | 35 | Atypical | II | Convexity/Parasagittal | Severe (+++) | No | MFC/GEP |
| 13 | -(14/22/X) | F | 76 | Meningothelial | I | Convexity/Parasagittal | No | No | MFC/GEP |
| 16A | -(1p/14/22) +9 | F | 60 | Transitional | I | Convexity | Moderate (++) | Yes | MFC/GEP |
| 16B | -(1p/14/22) | F | 62 | Atypical | II | Convexity | Moderate (++) | Yes | GEP |
| 18 | - (1/10/14/15/17/18/22/X) | F | 52 | Atypical | II | Convexity/Parasagittal | Moderate (++) | No | MFC/GEP |
| 19 | -(1p/14/22/X) +1q | F | 70 | Transitional | I | Cranial base | Moderate (++) | No | MFC/GEP |
| 38 | - (1p/10/14/22/18) | F | 84 | Meningothelial | I | Convexity | Severe (+++) | No | MFC |
| 44 | +(1q/7/10/15/17/18/22/Y/X) | M | 68 | Meningothelial | I | Convexity/Parasagittal | Moderate (++) | No | MFC |
| 53 | -(14/22/Y) | M | 61 | Meningothelial | I | Convexity | Light (+) | No | MFC |
| 59 | -(1p/7/14/15/18/22) +1q | F | 76 | Transitional | I | Convexity | Moderate (++) | No | MFC |
| 61 | -(1p/18/22) +9 | M | 54 | Meningothelial | I | Convexity | No | No | MFC |
| 62 | +(1q/7/14/15/17) ++18 | M | 68 | Transitional | I | Parasagittal | Severe (+++) | No | MFC |
| 64 | -(1p/22) | M | 48 | Transitional | I | Parasagittal | Severe (+++) | No | MFC |
| 68 | -(1p/22/Y) +1q | M | 66 | Transitional | I | Convexity | Moderate (++) | No | MFC |
| 75 | -(1p/14/18/22) +1q | F | 72 | Transitional | I | Convexity | Moderate (++) | No | MFC |
| 73 | -1p | M | 66 | Meningothelial | I | Intraosseous | No | No | MFC |

WHO: World Health Organization; M: Male; F: Female; iFISH: interphase fluorescence *in situ* hybridization; Tumors with ≥2 chromosomal changes were classified as carrying complex karyotypes; GEP: Gene expression profiling, MFC: Multiparameter flow cytometry immunophenotyping.
